# Neutrophil extracellular traps: emerging drivers and therapeutic targets in abdominal aortic aneurysm pathogenesis

**DOI:** 10.3389/ebm.2025.10781

**Published:** 2026-01-07

**Authors:** Xinyi Lyu, Qi Liu, Jiahao Shi, Yajun Chen, Xianpeng Dai

**Affiliations:** 1 Department of Vascular Surgery, The Second Affiliated Hospital, Hengyang Medical School, University of South China, Hengyang, China; 2 Department of Endocrinology and Metabolism, The Second Affiliated Hospital, Hengyang Medical School, University of South China, Hengyang, China

**Keywords:** neutrophil extracellular traps, abdominal aortic aneurysm, pathogenesis, therapeutic targets, biomarkers

## Abstract

Abdominal aortic aneurysm (AAA) is a life-threatening condition with no effective pharmacological treatments, underscoring the critical need to identify novel therapeutic targets. Emerging translational and clinical evidence implicates neutrophil extracellular traps (NETs) as potential drivers of AAA pathogenesis. This review systematically delineates the mechanisms by which NETs contribute to aortic wall degradation, focusing on their direct cytotoxicity to vascular smooth muscle cells (VSMCs), induction of VSMC phenotypic switching and ferroptosis, amplification of inflammatory cascades, and propagation of thromboinflammation. Key mediators include PAD4, IL-1β, PI3Kγ, neutrophil elastase, myeloperoxidase, and mitochondrial DNA. NET components (citrullinated histone H3, cell-free DNA, neutrophil elastase) serve as promising diagnostic and prognostic biomarkers. Preclinical studies highlight the efficacy of NET-targeting strategies, including inhibiting NET formation, degrading existing NETs, neutralizing cytotoxic components, and modulating downstream pathways (e.g., with ferroptosis inhibitors). Nanotechnology platforms enhance site-specific delivery of these agents. By integrating the research background with its practical implications, we conclude that targeting NETs represents a promising paradigm shift. Despite translational challenges, this approach offers a rational framework for developing the first pharmacotherapies aimed at stabilizing AAA and addressing a major unmet clinical need.

## Impact statement

This review establishes neutrophil extracellular traps (NETs) as central drivers of abdominal aortic aneurysm (AAA) progression, a life-threatening condition lacking effective drug therapies. It synthesizes groundbreaking evidence showing how NETs actively degrade the aortic wall by killing vascular muscle cells, amplifying inflammation, and promoting blood clots within the aneurysm. Crucially, we identify NET components (e.g., citrullinated histone H3) as novel diagnostic and prognostic biomarkers linked to aneurysm growth and rupture risk. Furthermore, the work highlights promising NET-targeting therapeutic strategies—including inhibitors of NET formation, degraders of existing NETs, and microbiome modulators—that significantly reduce AAA progression in preclinical models. By defining NETs as fundamental mediators and actionable targets, this review provides a transformative framework for developing the first pharmacological interventions to stabilize AAA, addressing a major unmet clinical need.

## Introduction

Abdominal aortic aneurysm (AAA), a potentially life-threatening dilation of the infrarenal aorta, characterized by progressive inflammatory infiltration, extracellular matrix degradation, and vascular smooth muscle cell (VSMC) depletion [1–3]. With no effective pharmacotherapies available, rupture carries a high mortality rate [4–6]. Inflammation is a recognized cornerstone of AAA pathogenesis, which operates within a complex interplay of genetic predisposition, biomechanical stress, metabolic disease, and environmental factors such as smoking, and involves innate and adaptive immune cells [7–9]. Traditionally viewed as first responders, neutrophils contribute to host defense beyond phagocytosis via a distinct process known as Neutrophil extracellular trap (NET) formation, or NETosis [10–12]. NETs, released by neutrophils, are web-like structures composed of decondensed chromatin decorated with cytotoxic granular proteins such as myeloperoxidase (MPO), neutrophil elastase (NE), and citrullinated histone H3 (CitH3), which function to ensnare pathogens [13–15]. Emerging translational and clinical evidence implicates neutrophil extracellular traps (NETs) as potential driver of AAA progression. It is important to note, however, that while these associations are compelling, the causal role of NETs in human AAA pathogenesis is still being delineated, with much of the mechanistic insight derived from preclinical models. Components of NETs, such as CitH3, cell-free DNA (cfDNA), and NE, are significantly elevated in both the plasma and tissues of AAA patients, and these elevations correlate with aneurysm size, growth rate, and severity [16–18]. Histologically, NETs localize within the aortic wall and intraluminal thrombus (ILT), where they mediate multifaceted damage [19, 20].

This review synthesizes evidence establishing NETs as fundamental drivers of AAA pathogenesis. It will specifically examine the molecular and cellular mechanisms by which NETs cause ECM degradation, induce VSMC death (apoptosis, ferroptosis) and phenotypic switching, amplify inflammation (cytokine cascades, pDC activation), and drive metabolic/epigenetic dysregulation. Systemic factors (gut dysbiosis, thromboinflammation) amplifying NETosis are explored. The translational potential of NET components as biomarkers and emerging NET-targeting therapeutic strategies are critically evaluated. Finally, challenges in translating these discoveries to meet the unmet clinical need in AAA management are addressed.

## Categorization and mechanisms of NETosis

NETosis can be classified by stimulus (microbial vs. sterile), cellular outcome (lytic/suicidal vs. vital), and molecular pathway (NADPH oxidase-dependent vs. -independent). In the context of AAA, sterile inflammation-induced and thromboinflammatory NETosis are particularly relevant, driven by DAMPs, cytokines, and platelet-neutrophil interactions. A detailed summary of NETosis classifications is provided in Supplementary Table S1.

### Pathological mechanisms of NETosis in AAA

NETosis is a central driver of AAA pathogenesis. Its detrimental effects unfold through distinct but interconnected mechanisms, categorized as follows (Table 1).

**TABLE 1:** Pathological mechanisms of NETosis in AAA.

Category	Key elements	Pathological effects in AAA	Experimental evidences
Molecular triggers	IL-1β: induces ceramide synthase 6 → C16-ceramide → nuclear changes → NETosis	Promotes neutrophil activation and NET release, driving initial inflammation and wall damage	Il1b KO mice show reduced AAA [21]
	oxLDL: Potent NETosis inducer	Links dyslipidemia to vascular inflammation and NET-driven injury	Plasma oxPL/apoB levels correlate with citH3 levels in AAA patients [22]
	PDK isoenzymes: upregulated in AAA → increases lactate/p-PDH → metabolic shift	Drives NETosis and vascular inflammation	DCA (PDK inhibitor) reduces NETosis in mouse models [23]
Cellular damage	NET components (NE, MPO, CitH3): Direct proteolytic attack on ECM (elastin, collagen)	Elastin degradation, ECM fragmentation → loss of structural integrity	NETs increase MMP-2/9 activity [24]; NE/MPO directly degrade elastin [20, 25]
	VSMC phenotypic switching: NETs suppress Hippo-YAP pathway → increases synthetic/pro-inflammatory VSMCs	VSMC loss of contractility → impaired repair; increases inflammation	*Padi4* or *Yap* knockout (KO) prevents VSMC transformation [26]
	VSMC ferroptosis: NETs deplete mitochondrial glutathione (reduces SLC25A11) + inhibit PI3K/AKT → iron-dependent death	Massive VSMC loss → wall thinning, rupture risk	Ferroptosis inhibitors reduce AAA in mouse models [27, 28]
Systemic drivers	Gut dysbiosis: reduces *Ruminococcus intestinalis* → reduces butyrate	Increases neutrophil infiltration → increases NOX2-dependent NETosis → aortic dilation	Butyrate supplementation or *Ruminococcus intestinalis* gavage attenuates AAA [29]
	Thromboinflammation: NETs → activate pDCs → increases type I IFNs → increases inflammation	Sustained inflammation, macrophage activation, and ECM degradation	pDC depletion or type I IFN blockade attenuates AAA [20]
	PAD4: Mediates histone citrullination → chromatin decondensation → NETosis	Essential for NET formation; drives VSMC apoptosis and ECM damage	PAD4 inhibitors or KO reduces VSMC apoptosis and AAA rupture [18, 30, 31]
	PI3Kγ: Activates non-canonical pyroptosis → NETosis	Amplifies neutrophil-driven inflammation and wall injury	PI3Kγ inhibition reduces NETs in AAA models [32]
Thrombus interface	NET Reservoir: ILT accumulates high levels of citH3, NE, MPO, and cfDNA.	Creates hyper-inflammatory/proteolytic niche → recruits neutrophils → perpetuates NETosis	Thrombus contains 30× more citH3 than wall, CXCL1/CXCL8 recruit neutrophils [10, 19]
	pDC activation: ILT-concentrated NETs → robust pDC activation → increases type I IFNs	Amplifies vascular inflammation and ECM breakdown	pDCs activated by NETs in ILT [20]

Key Abbreviations: citH3, Citrullinated Histone H3; DCA, Dichloroacetate; ECM, Extracellular Matrix; IFN, Interferon; IL-1β: Interleukin-1 beta; ILT, Intraluminal Thrombus; MMP, Matrix Metalloproteinase; MPO, Myeloperoxidase; NE, Neutrophil Elastase; NETosis, Neutrophil Extracellular Trap formation; oxLDL, oxidized low-density lipoprotein; PAD4, Peptidyl Arginine Deiminase 4; pDC, plasmacytoid Dendritic Cell; PDK, Pyruvate Dehydrogenase Kinase; PI3K/AKT, Phosphoinositide 3-kinase/Protein Kinase B; PI3Kγ, Phosphoinositide 3-Kinase gamma; SLC25A11, Mitochondrial Glutamate Carrier; VSMC, Vascular Smooth Muscle Cell.

### Molecular triggers of NETosis

Key molecular mediators of NETosis in AAA include interleukin-1β (IL-1β). IL-1β induces ceramide synthase 6-mediated synthesis of C16-ceramide, thereby promoting the nuclear changes essential for NET formation [21]. Oxidized low-density lipoprotein (oxLDL) is another potent inducer of NETosis [22], mechanistically linking dyslipidemia to vascular inflammation in AAA. Additionally, in human AAA tissue PDK isoenzymes are upregulated, which drives a metabolic shift characterized by increased lactate production and phosphorylated PDH levels [23]. Crucially, experimental inhibition of PDK not only reduces NETosis but also attenuates AAA pathogenesis in *in vivo* and *in vitro* models, confirming its significant role [23].

### Cellular damage by NET components

NETs, which are composed of chromatin filaments complexes with cytotoxic proteins such as NE, MPO, and CitH3 [20, 24, 25], directly inflict damage upon the vascular wall. Beyond direct injury, NETs significantly exacerbate vascular pathology by inducing phenotypic switching and dysfunction in VSMCs [33]. Specifically, neutrophil elastase (NE), a key component of NETs, has been identified as a direct mediator that suppresses the Hippo-YAP signaling pathway, promoting the transition of VSMCs towards a synthetic, pro-inflammatory phenotype. This shift is associated with distinct histone modifications—enrichment of H3K4me3 and reduction of H3K27me3—at the promoters of contractile apparatus genes [26].

Notably, NETs are potent inducers of VSMC ferroptosis, an iron-dependent form of regulated cell death [27]. Mechanistically, the proteolytic activity of NET-associated NE contributes to the degradation of the mitochondrial glutamate carrier SLC25A11, leading to glutathione depletion and compromising cellular antioxidant defenses [27]. Concurrently, NETs inhibit the pro-survival PI3K/AKT signaling pathway, which downregulates the expression of the central anti-ferroptotic regulator, glutathione peroxidase 4 (GPX4), thereby synergistically enhancing VSMC susceptibility to ferroptosis [28].

### Systemic drivers amplifying NETosis

#### Gut microbiome dysregulation

Alterations in the gut microbiota composition (dysbiosis), particularly a reduced abundance of *Ruminococcus intestinalis*, contribute significantly to AAA pathogenesis by influencing NETosis. This dysbiosis diminishes microbial production of the short-chain fatty acid butyrate. Butyrate deficiency, in turn, promotes neutrophil infiltration into the aortic wall and enhances NOX2-dependent NET formation, potentially through mechanisms involving the inhibition of histone deacetylases (HDAC) activity and subsequent suppression of pro-inflammatory signaling pathways, ultimately accelerating aortic dilation [29].

#### Thromboinflammation and feed-forward loops

NETosis actively fuels a pro-thrombotic environment within the aneurysm sac. NET components, notably cfDNA and CitH3, stimulate plasmacytoid dendritic cells (pDCs). Activated pDCs produce type I interferons (IFNs), which sustain vascular inflammation and promote macrophage activation. Furthermore, NETs facilitate the development of intraluminal thrombus. Critically, this thrombus acts as a reservoir for NET components and associated proteases, creating a potent pro-inflammatory and proteolytic microenvironment. This niche further recruits neutrophils, perpetuating NETosis and driving continuous wall degradation. Critically, the presence of thrombus significantly enhances the efficacy of NETosis inhibitors in reducing AAA progression in experimental models, highlighting the centrality of this thromboinflammatory cycle [20, 23].

#### Epigenetic and transcriptional regulation

Key molecular regulators underpin NETosis in AAA. PAD4-mediated histone citrullination is an essential step for chromatin decondensation during NET formation. Pharmacological inhibition or genetic deletion of PAD4 markedly reduces NET generation, attenuates VSMC apoptosis, and decreases AAA rupture incidence [18, 30, 31]. Additionally, phosphoinositide-3-kinase γ (PI3Kγ) signaling promotes NETosis by activating non-canonical pyroptosis pathways dependent on cAMP/PKA signaling [32].

## The thrombus interface: a critical hub in AAA pathogenesis

NETs actively promote the formation of ILT within AAA. Once established, the ILT functions as a dynamic repository, accumulating high concentrations of NET-derived components, such as DNA, CitH3, NE, MPO, and various proteases [10, 34]. This thrombotic niche fosters a potent pro-inflammatory microenvironment. Through the release of neutrophil-attracting chemokines, such as CXCL1 and CXCL8, the ILT recruits additional neutrophils to the site [19, 35]. This sustained neutrophil influx perpetuates NETosis and drives continuous degradation of the vascular wall. Notably, the ILT concentrates NET components to extraordinary levels. For instance, CitH3 accumulates within the thrombus at concentrations up to 30-fold higher than those found in the adjacent aortic wall [13, 18]. This concentrated reservoir serves as a potent platform for activating pDCs, stimulating robust type I IFN production, and thereby amplifying vascular inflammation [36].

The central role of the ILT in driving NETosis-dependent pathology is underscored by the differential efficacy of NETosis inhibitors. Pharmacological agents targeting NET formation, such as PAD4 inhibitors, demonstrate significantly enhanced efficacy in attenuating AAA progression in experimental models possessing an ILT [27, 31]. These highlight the ILT not merely as a pathological feature, but as a crucial therapeutic target within the NETosis-amplifying cascade of AAA.

### NETs as biomarkers in AAA

NET components exhibit compelling potential as clinical biomarkers across the entire AAA disease continuum. Their emerging diagnostic and prognostic utility, supported by recurrent evidence in the literature, is systematically summarized below.

### Circulating NET components serve as diagnostic and prognostic biomarkers

Consistently elevated levels of specific NET components are detected in the plasma/serum of AAA patients compared with healthy controls and patients with other vascular pathologies, such as peripheral artery disease (PAD). These biomarkers demonstrate significant diagnostic and prognostic utility.

#### CitH3

A highly specific NETosis marker with significantly increased concentrations in AAA plasma and tissue—particularly within the ILT. It demonstrates diagnostic potential (AUC ≈0.705), predicts short-term AAA progression (e.g., 6-month growth), and decreases markedly following successful surgical repair, highlighting its value for postoperative monitoring [10, 16–18, 31, 37].

#### cfDNA

As the structural DNA backbone of NETs, plasma cfDNA levels are substantially elevated in AAA patients. These elevations correlate strongly with established markers of neutrophil activation and NET formation, highlighting the central role of neutrophil dysregulation and NETosis in AAA progression and positioning cfDNA as a potential biomarker reflecting this key pathological pathway [10, 13, 16–18, 30, 37].

#### MPO & NE

These granular proteins, embedded within NET structures, are elevated in AAA plasma and tissue. They co-localize with NETs and correlate with both disease presence and activity, positioning them not only as specific markers of NET burden but also as potential indicators of disease severity and future risk [7, 10, 17, 20, 31, 37].

#### Oxidized DNA

Reflecting NETosis-associated oxidative stress. Specific methodologies (e.g., immunoprecipitation followed by qPCR) enable differentiation between oxidized nuclear and mitochondrial DNA in plasma. Studies show that AAA patients exhibit trends toward increased oxidized mitochondrial DNA and enrichment of mitochondrial DNA within the oxidized fraction, suggesting a potential link between NETosis and specific organellar damage pathways [16].

#### Multiplex biomarker panels

Combinations of CitH3, cfDNA, MPO, and potentially oxidized phospholipids on apolipoprotein B-100 (oxPL/apoB) demonstrate enhanced diagnostic accuracy and progression risk stratification over single-marker approaches [17, 18, 30].

## Association with disease severity and progression

Circulating levels of key NET biomarkers—including CitH3, cfDNA, MPO, and NE—show strong correlations with established AAA risk factors and adverse clinical outcomes. Specifically, these biomarkers demonstrate significant associations with both AAA maximum diameter and annual expansion rate [16–18, 30, 37]. It is worth noting that Eilenberg et al. reported that elevated CitH3 levels are a predictive indicator for accelerated AAA growth [18].

NET components are consistently found at significantly higher concentrations within the AAA wall tissue and, in particular, are localized within the ILT [18–21, 26, 31, 37]. Furthermore, advances in molecular profiling, utilizing machine learning and multi-omics analyses, have identified distinct NET-related gene expression signatures (e.g., involving *DUSP26, FCN1, MTHFD2, GPRC5C, SEMA4A, CCR7*). These signatures hold considerable diagnostic potential as novel tools for predicting the likelihood and trajectory of AAA progression [33].

### Link to pathogenic mechanisms and comorbidities

Circulating NET biomarker levels serve as indicators of key pathological processes underlying AAA development and progression.

#### Inflammation

NET biomarker concentrations exhibit strong correlations with both general systemic inflammatory markers and specific cytokines (e.g., IL-1β, IL-6) known to be critically involved in AAA pathogenesis [4, 7, 21]. This underscores the integral role of NETs within the inflammatory cascade driving AAA.

#### Oxidative stress

The significant correlation observed between oxPL/apoB (a biomarker reflecting oxidized phospholipids on apolipoprotein B-100 particles) and CitH3 suggests that oxidized lipids actively promote NETosis within the AAA milieu [17]. This process may constitute a significant mechanism contributing to disease progression.

#### VSMC dysfunction

NETs directly contribute to AAA wall weakening by inducing detrimental changes in VSMCs, including phenotypic switching, apoptosis, senescence, and ferroptosis. Consequently, elevated NET biomarker levels may indirectly reflect the extent of this crucial VSMC damage [21, 26–28].

#### Gut microbiome dysbiosis

Alterations in the gut microbiota composition associated with AAA, such as reduced abundance of *Roseburia intestinalis*, have been linked to enhanced NET formation. Therefore, specific NET biomarker profiles may potentially reflect these underlying dysbiotic states [29].

## Potential for monitoring therapeutic interventions

NET-derived biomarkers demonstrate significant promise as tools for monitoring responses to therapeutic interventions in AAA. Pharmacological strategies targeting NETosis—including PAD4 inhibitors (e.g., YW3-56), DNase1, resolvin D1, and PDK inhibitors (e.g., dichloroacetate, DCA)—consistently attenuate AAA progression in preclinical models. This therapeutic efficacy is paralleled by a measurable reduction in circulating NET biomarker levels, such as CitH3, cfDNA, and NE [5, 18, 19, 21, 23, 28, 31, 38]. Clinically, CitH3 levels decrease significantly following successful surgical AAA repair, underscoring their potential utility in tracking post-interventional outcomes [18]. Furthermore, emerging NET-targeting nanomedicine approaches (e.g., GlycoRNA nanoparticle-delivered siMT1 [GlycoRNA-NP-siMT1], lactoferrin-coated calcium dipicolinate nanoparticles [LaCD NP]) effectively reduce disease progression in preclinical AAA models [5, 38]. In these studies, the concomitant reduction in NET biomarkers serves as a critical quantitative readout for assessing treatment efficacy.

Despite these promising diagnostic and prognostic associations, the clinical translation of NET-derived biomarkers faces several challenges. Circulating levels of markers such as CitH3, cfDNA, MPO, and NE show marked heterogeneity across studies, influenced by variations in sample processing, detection methodologies, and patient cohort characteristics. The lack of standardized, validated assays and universally accepted clinical cut-off values currently prevents their routine application in clinical decision-making. Therefore, large-scale, multi-center prospective studies are crucial to harmonize detection protocols and definitively establish the utility of these biomarkers for risk stratification and monitoring therapeutic responses.

### Imaging biomarkers

Beyond circulating molecular biomarkers, emerging molecular imaging techniques offer the potential for direct, non-invasive visualization of NETosis *in vivo*. A promising approach utilizes anti-Ly6G antibody-conjugated superparamagnetic iron oxide nanoparticles (Ly6G-SPIONs) in conjunction with Magnetic Particle Imaging (MPI). This combined platform enables highly sensitive detection and quantification of neutrophil infiltration specifically within AAA lesions in murine models. Critically, the MPI signal intensity correlates strongly with AAA severity and exhibits a significant decrease following pharmacological inhibition of NETosis, demonstrating its utility as a dynamic imaging biomarker for disease activity and therapeutic response [37].

### Therapeutic targeting of NETs in AAA

NETs have been established as key pathogenic drivers of AAA progression, orchestrating vascular inflammation, VSMC death, extracellular matrix degradation, and maladaptive vascular remodeling. These processes collectively weaken the aortic wall, ultimately driving aneurysm expansion and rupture risk. Therefore, pharmacologically targeting NET formation or activity has emerged as a promising therapeutic approach to mitigate AAA pathogenesis, as summarized in Table 2.

**TABLE 2:** Therapeutic targeting of NETs in AAA.

Therapeutic strategy	Molecular target	Intervention	Mechanism	Experimental outcomes	References
PAD4 inhibition	PAD4	Genetic deletion of *Padi4*; pharmacological inhibitors (e.g., Cl-amidine, YW3-56)	Inhibits histone citrullination and NET formation	Reduces NET release; reduces AAA formation/rupture; increases SMC contractility; reduces plasma citH3 (biomarker)	[4, 18, 19, 26, 31]
Targeting NET components	MT1	GlycoRNA-NP-siMT1 nanoparticles	Coated NPs deliver MT1 siRNA to AAA site. Competitively inhibits neutrophil infiltration & suppresses MT1 expression, inhibiting NET formation	Reduces NET formation; reduces pathological remodeling; reduces aortic dilation	[5]
	DPPI/NE/PR3	Genetic deficiency; pharmacological inhibition	DPPI activates NE/PR3, crucial for NET release. NE degrades DNA during NETosis	Protects against AAA development	[20]
Immunometabolic reprogramming	PDK	DCA; PDK1-siRNA	Corrects the skewed PDK/PDH axis metabolism	Reduces NET release; reduces neutrophil infiltration; increases SMC contractile phenotype; prevents elastin breakdown; reduces AAA formation (∼58%)	[23]
PI3Kγ pathway inhibition	PI3Kγ	PI3Kγ inhibitors	Acts upstream to promote NET formation through noncanonical pyroptosis (cAMP/PKA-dependent)	Reduces NETosis; reduces aortic wall inflammation; ameliorates AAA	[32]
Pro-resolving mediators	CerS6/C16-ceramide	RvD1	Inhibits CerS6/C16-ceramide synthesis, reducing NETosis	Reduces NETosis (reduces CitH3, reduces NE); reduces AAA formation (elastase & Ang II models); reduces inflammation (e.g., IL-1β); reduces MMP activity	[19]
Anti-inflammatory nanotherapies	General neutrophil inflammation/NETosis	LaCD nanoparticles	Intrinsically anti-inflammatory NPs accumulate in aneurysmal aorta, inhibiting neutrophil-mediated inflammation and NETosis	Reduces NET formation; suppresses NET-driven inflammation & pathological remodeling; reduces AAA	[38]
Targeting NET-induced SMC pathologies	mitoGSH depletion/ferroptosis	Ferrostatin-1; prevent mitoGSH depletion	NETs induce SMC ferroptosis via reduces mitoGSH (destabilizing SLC25A11)	Protects against AAA	[27]
	SLC25A11/ferroptosis	MSC-EVs	Reduce NET release and inhibit NET-induced SMC ferroptosis	Alleviates AAA	[28]
	Hippo-YAP pathway/H3K4me3/H3K27me3	Potential target implied	NETs promote synthetic/proinflammatory SMC phenotypes via inhibiting Hippo-YAP and modulating histone marks (H3K4me3/H3K27me3) at gene promoters		[26]
Microbiome modulation	Gut dysbiosis/Butyrate levels	*Roseburia intestinalis* supplementation; butyrate	Corrects dysbiosis, increases butyrate. Reduces NOX2-dependent NETosis	Reduces neutrophil infiltration; reduces NETosis; reduces inflammation; reduces SMC phenotypic switching; reduces AAA	[29]

Key Abbreviations: AAA, Abdominal Aortic Aneurysm; Ang II, Angiotensin II; cAMP, Cyclic Adenosine Monophosphate; CerS6, Ceramide Synthase 6; citH3, Citrullinated Histone H3; DCA, Dichloroacetate; DPPI, Dipeptidyl Peptidase I; EVs, Extracellular Vesicles; Hippo-YAP, Hippo pathway and Yes-Associated Protein; IL-1β, Interleukin-1 beta; mitoGSH, Mitochondrial Glutathione; MMP, Matrix Metalloproteinase; MSC, Mesenchymal Stem Cell; MT1, Metallothionein 1; NE, Neutrophil Elastase; NETs, Neutrophil Extracellular Traps; NOX2, NADPH Oxidase 2; NP, Nanoparticle; PAD4, Peptidyl Arginine Deiminase 4; PDH, Pyruvate Dehydrogenase; PDK, Pyruvate Dehydrogenase Kinase; PI3Kγ, Phosphoinositide 3-Kinase gamma; PKA, Protein Kinase A; PR3, Proteinase 3; RvD1, Resolvin D1; siRNA, small interfering RNA; SLC25A11, Mitochondrial Glutamate Carrier; SMC, Smooth Muscle Cell.

### PAD4 inhibition

PAD4 catalyzes histone citrullination, a critical step in NET formation [39, 40]. Consequently, PAD4 represents a pivotal therapeutic target for NET-driven pathologies like AAA. Both genetic ablation of *Padi4* and pharmacological inhibition with compounds such as Cl-amidine or YW3-56 significantly suppress NET release in preclinical AAA models. This suppression translates to substantial therapeutic benefits, including attenuated AAA formation and rupture risk, alongside preserved VSMCs contractility [4, 18, 19, 26, 31]. Importantly, plasma levels of citrullinated CitH3, a direct product of PAD4 activity and a specific NET biomarker, correlate with both AAA presence and aneurysm growth rate. Furthermore, CitH3 levels decrease significantly following successful surgical AAA repair. These findings not only highlight the utility of CitH3 as a sensitive biomarker for disease activity and therapeutic response but also provide compelling clinical validation for PAD4 as a viable therapeutic target in AAA [18].

## Targeting specific NET components

Metallothionein 1 (MT1) is significantly upregulated within human and murine AAA lesions, where it actively promotes NET formation [5]. To counteract this, glycoRNA-conjugated nanoparticles delivering MT1-targeting siRNA (GlycoRNA-NP-siMT1) achieve site-specific delivery to aneurysmal tissue. This nanotherapeutic platform effectively suppresses NET generation, pathological vascular remodeling, and aortic dilation through dual mechanisms: competitively inhibiting neutrophil infiltration while directly silencing pathogenic MT1 expression within inflammatory cells [5]. Parallel strategies target the proteolytic cascade centered on dipeptidyl peptidase I (DPPI/Cathepsin C), which activates NE and PR3 to drive NET release. Genetic deficiency in either DPPI or its downstream effectors confers protection against experimental AAA, confirming their non-redundant role in NET-driven pathogenesis [20]. Crucially, NE executes dual functions in this process—facilitating NET chromatin decondensation through histone degradation while independently contributing to extracellular matrix destruction within the aortic wall [5, 20].

### Immunometabolic reprogramming

Targeting the dysregulated PDK/pyruvate dehydrogenase (PDK/PDH) axis through inhibition of PDK—using either DCA or PDK1-targeting siRNA—is a potent immunometabolic intervention for AAA. This metabolic reprogramming effectively suppresses NET release and reduces pathological neutrophil infiltration into the aortic wall. Concurrently, it preserves the contractile phenotype of SMCs and prevents elastin degradation. Collectively, these mechanisms attenuate AAA progression by approximately 58%, as evidenced by significant reduction in aortic dilation in preclinical models [23].

### PI3Kγ pathway inhibition

PI3Kγ functions as an upstream regulator of NET formation in AAA, driving this process through a noncanonical pyroptosis pathway dependent on cAMP/PKA signaling activation. Pharmacological inhibition of PI3Kγ significantly reduces NETosis, attenuates inflammatory cell infiltration within the aortic wall, decreases pro-inflammatory cytokine production, and ultimately ameliorates key AAA pathological features including vascular remodeling and aneurysm expansion in preclinical models [32].

### Resolvin D1 (RvD1)

RvD1, a specialized pro-resolving lipid mediator derived from docosahexaenoic acid (DHA), potently suppresses NET formation by inhibiting ceramide synthase 6 (CerS6)-dependent C16-ceramide biosynthesis. This molecular intervention significantly reduces key NET components including CitH3 and NE. Therapeutic administration of RvD1 attenuates AAA progression in both elastase-perfusion and angiotensin II (Ang II)-induced murine models. The protective effects are mediated through substantial reductions in pro-inflammatory cytokines (e.g., IL-1β) and matrix metalloproteinase (MMP-2/MMP-9) activity, collectively preserving vascular structural integrity [19].

### Anti-inflammatory nanotherapies

Engineered nanoparticles with inherent anti-inflammatory properties, exemplified by lactoferrin-coated calcium dipicolinate nanoparticles (LaCD NPs), demonstrate targeted biodistribution to aneurysmal aortic tissue. This nanotherapeutics effectively suppresses neutrophil-mediated inflammation through inhibition of NLRP3 inflammasome activation and subsequent IL-1β release. Crucially, they potently attenuate NETosis, thereby interrupting the self-perpetuating cycle of NET-driven inflammation. This dual-action mechanism significantly reduces pro-inflammatory cytokine cascades and pathological vascular remodeling, establishing LaCD NPs as promising targeted therapeutics for abdominal aortic aneurysm intervention [38].

### Targeting NET-Induced SMC pathologies

NETs drive SMC ferroptosis by destabilizing the mitochondrial glutamate carrier SLC25A11, resulting in depletion of mitochondrial glutathione (mitoGSH). This metabolic disruption compromises cellular redox homeostasis and promotes iron-dependent cell death. Therapeutic prevention of mitoGSH depletion or pharmacological inhibition of ferroptosis pathways—using specific inhibitors such as ferrostatin-1—confers significant protection against AAA development in preclinical models [27]. Additionally, mesenchymal stem cell-derived extracellular vesicles (MSC-EVs) represent a complementary approach, as they simultaneously suppress NET release and directly inhibit NET-induced SMC ferroptosis, thereby attenuating AAA progression [28]. Beyond ferroptosis, NETs additionally promote pathological SMC phenotypic switching toward synthetic and proinflammatory states. This transition is mechanistically linked to NET-mediated inhibition of the Hippo-YAP signaling pathway and epigenetic dysregulation through altered histone methylation marks (specifically reduced H3K4me3 and elevated H3K27me3) at promoters of contractile apparatus genes [26].

### Microbiome modulation

Gut dysbiosis represents a significant pathogenic contributor to AAA development, characterized by depletion of *Roseburia intestinalis* and reduced butyrate production. Therapeutic restoration of microbial homeostasis through *Roseburia intestinalis* supplementation or direct butyrate administration effectively suppresses neutrophil infiltration into the aortic wall, inhibits NOX2-dependent NETosis, attenuates pro-inflammatory cytokine cascades, and prevents pathological SMC phenotypic switching. These coordinated mechanisms collectively reduce aortic dilation by >50% in preclinical models, demonstrating the therapeutic potential of microbiome modulation in AAA management [29].

## Discussion

The clinical translation of NET-targeting therapies faces significant hurdles. Current preclinical models—particularly acute angiotensin II infusion in mice which induces AAA over weeks—fail to adequately recapitulate the slow, smoldering inflammation that characterizes human AAA progression over years or decades [41–43]. Further complicating translation, the therapeutic efficacy of many NET inhibitors is thrombus-dependent, necessitating validation in models that faithfully mimic human disease progression timelines.

Therapeutic specificity and safety remain paramount. Furthermore, the therapeutic suppression of NETosis raises legitimate concerns regarding the impairment of innate antimicrobial defense. Systemic inhibition of neutrophil function, for instance via PAD4 blockade or DNase administration, could potentially increase susceptibility to infections. Therefore, achieving cell- and context-specificity—for example, through localized delivery systems such as nanoparticles or the development of inhibitors targeting disease-specific NET components—is paramount to maximizing safety. Patient stratification based on infection risk and immunocompetence will also be a crucial consideration for future clinical application. While PAD4 inhibitors show promise due to functional redundancy in host immunity, targeting downstream effectors (e.g., SMC ferroptosis pathways) may offer superior safety profiles.

NET heterogeneity—driven by stimulus-specific activation, disease stage, and anatomically distinct microenvironments—demands precision interventions tailored to individual pathological contexts [44–46]. Validation of NET biomarkers (e.g., CitH3, cfDNA) through large-scale longitudinal clinical studies is imperative for robust diagnostic and prognostic applications. Spatially resolved multi-omics approaches combined with advanced molecular imaging (e.g., Ly6G-NP-MPI) will enable comprehensive mapping of NET spatiotemporal dynamics within AAA microenvironments.

Future success will require: (1) Combinatorial strategies concurrently targeting NETs and complementary pathogenic pathways (e.g., MMPs, cytokine networks, renin-angiotensin signaling), (2) Prioritization of interventions for established aneurysms rather than prevention-only paradigms, and (3) Identification of NET-driven patient endotypes (e.g., high-CitH3 phenotypes, rapid progressors) to enable personalized therapeutic stratification.

The promising preclinical data now necessitate a decisive transition to clinical validation. Future success is contingent upon initiating early-phase human trials to evaluate the safety, tolerability, and bioactivity of NET-inhibiting agents. These studies should prioritize patient populations with a high unmet need, such as those with rapidly expanding aneurysms, and utilize the emerging NET-related biomarkers (e.g., plasma CitH3) for patient enrichment and pharmacodynamic assessment.

## Summary

NETs are emerging as key regulators within the multifactorial network of AAA pathogenesis, integrating neutrophil activation with SMC death, ECM degradation, inflammation, thrombosis, and metabolic reprogramming. Their consistent detection in human AAA tissues and circulation, coupled with strong correlations to disease severity and expansion, underscores their clinical relevance. Preclinical strategies targeting NET formation, degradation, and their downstream effects demonstrate robust efficacy in attenuating AAA progression, with nanotechnology offering promising avenues for enhanced site-specific delivery.

Despite this promise, translating these findings necessitates focused future efforts. First, the clinical utility of NET-derived biomarkers requires validation in large-scale, longitudinal cohorts to establish standardized thresholds for risk stratification. Second, the refinement of targeted delivery systems is crucial to maximize therapeutic efficacy while minimizing systemic impact on host defense. Third, combination therapies concurrently targeting NETs and complementary pathways (e.g., MMPs, renin-angiotensin system) should be explored to achieve synergistic effects in established aneurysms. Finally, identifying NET-driven patient endotypes will be essential for personalizing therapeutic interventions. By addressing these priorities, NETosis inhibition can evolve from a compelling preclinical concept into a viable clinical strategy for stabilizing AAA.
