# Supplementary material for: Neutrophil extracellular traps: emerging drivers and therapeutic targets in abdominal aortic aneurysm pathogenesis
Source: Exp Biol Med (Maywood). 2026 Jan 7;250:10781. doi: 10.3389/ebm.2025.10781 (PMC12822572; doi:10.3389/ebm.2025.10781)
Supplement: Supplementary file 1 [file Table1.docx]

**Supplementary Table 1 Classification of NETosis**

| **Classification Framework** | **Category** | **Key Characteristics** | **Molecular Mechanisms/Triggers** | **Pathophysiological Contexts & Examples** | **Refs** |
| --- | --- | --- | --- | --- | --- |
| **Cell Fate** | **Suicidal NETosis** | Programmed neutrophil lysis; cell death-dependent | NOX-dependent ROS burst → PAD4 activation → chromatin decondensation →extrusion of nuclear DNA release with MPO/NE | Classic antimicrobial defense; tissue injury amplifier | [^1-8^](#_ENREF_1) |
|  | **Vital NETosis** | Preserves viability & partial function; cell death-independent | NOX-independent; TLR activation (e.g., by HMGB1/cytokines) → mtDNA/nuclear DNA release | Rapid pathogen containment; minimal bystander damage | [^5^](#_ENREF_5)^,^ [^8-10^](#_ENREF_8) |
| **Stimulus Type** | **Microbial-Induced** | Pathogen-triggered | PAMPs (e.g., LPS, viral RNA) | Bacterial/fungal/viral infections | [**^11-14^**](#_ENREF_11) |
|  | **Sterile Inflammation-Induced** | Non-infectious triggers | DAMPs (e.g., HMGB1, amyloid-β, IL-8/TNF-α, immune complexes) | Stroke, vasculitis, autoimmunity | [**^15-18^**](#_ENREF_15) |
| **Molecular Pathway** | **NOX/ROS-Dependent** | Canonical pathway; inhibitable by NOX/ROS blockade | ROS → PAD4 → autophagy → nuclear decondensation | Most infection/stress responses | [**^5^**](#_ENREF_5)**^,^** [**^19^**](#_ENREF_19)**^,^** [**^20^**](#_ENREF_20) |
|  | **NOX/ROS-Independent** | Resistant to classical inhibitors | Calcium flux, mtROS, complement activation | Alternative inflammatory cascades | [^17^](#_ENREF_17)^,^ [^21^](#_ENREF_21) |
| **Disease-Associated Phenotype** | **Thromboinflammatory** | Platelet-neutrophil interactions; fibrin-NET microthrombi | HMGB1-TLR4 signaling; platelet-derived DAMPs | COVID-19, APS, diabetic vasculopathy, stroke | [^9^](#_ENREF_9)^,^ [^17^](#_ENREF_17)^,^ [^22-27^](#_ENREF_22) |
|  | **Autoimmune-Potentiating** | Impaired clearance → autoantigen exposure (e.g., citH3, MPO/PR3) | Impaired DNase activity; NETophagy impairment | RA, SLE, ANCA-associated vasculitis | [^28-30^](#_ENREF_28) |
|  | **Cancer-Associated** | Promotes metastasis & therapy resistance | NET-mediated ECM remodeling; and reactivation of dormant tumor cells | Lung adenocarcinoma, breast cancer | [^31-33^](#_ENREF_31) |

**Key Abbreviations:** ANCA: Anti-Neutrophil Cytoplasmic Antibody; APS: Antiphospholipid Syndrome; DAMPs: Damage-Associated Molecular Patterns; ECM: Extracellular Matrix; HMGB1: High Mobility Group Box 1; IL-8: Interleukin-8; LPS: Lipopolysaccharide; mtDNA: Mitochondrial DNA; NE: Neutrophil Elastase; NETosis: Neutrophil Extracellular Trap formation; NETophagy: Impaired NET clearance via autophagy; NOX: NADPH Oxidase; PAD4: Peptidyl Arginine Deiminase 4; PAMPs: Pathogen-Associated Molecular Patterns; PR3: Proteinase 3; RA: Rheumatoid Arthritis; ROS: Reactive Oxygen Species; SLE: Systemic Lupus Erythematosus; TLR: Toll-Like Receptor; TNF-α: Tumor Necrosis Factor-alpha.

**References**

1. Brinkmann V, Reichard U, Goosmann C, Fauler B, Uhlemann Y, Weiss DS, Weinrauch Y, Zychlinsky A. Neutrophil extracellular traps kill bacteria. *Science* 2004;**303**:1532-5. <https://dx.doi.org/10.1126/science.1092385>

2. Thiam HR, Wong SL, Wagner DD, Waterman CM. Cellular Mechanisms of NETosis. *Annual review of cell and developmental biology* 2020;**36**:191-218. <https://dx.doi.org/10.1146/annurev-cellbio-020520-111016>

3. Zhu YP, Speir M, Tan Z, Lee JC, Nowell CJ, Chen AA, Amatullah H, Salinger AJ, Huang CJ, Wu G, Peng W, Askari K, Griffis E, Ghassemian M, Santini J, Gerlic M, Kiosses WB, Catz SD, Hoffman HM, Greco KF, Weller E, Thompson PR, Wong LP, Sadreyev R, Jeffrey KL, Croker BA. NET formation is a default epigenetic program controlled by PAD4 in apoptotic neutrophils. *Science advances* 2023;**9**:eadj1397. <https://dx.doi.org/10.1126/sciadv.adj1397>

4. Souza FW, Miao EA. Neutrophils only die twice. *Science advances* 2023;**9**:eadm8715. <https://dx.doi.org/10.1126/sciadv.adm8715>

5. Zambrano F, Uribe P, Schulz M, Hermosilla C, Taubert A, Sanchez R. Antioxidants as Modulators of NETosis: Mechanisms, Evidence, and Therapeutic Potential. *International journal of molecular sciences* 2025;**26**<https://dx.doi.org/10.3390/ijms26115272>

6. Signa S, Bartolucci M, Bonacini M, Bertoni A, Del Zotto G, Corcione A, Petretto A, Della Bella S, Bertelli R, Di Silvestre D, Lomagno A, Mauri P, Caorsi R, Bruschi M, Balin S, Bocca P, Volpi S, Catanoso MG, Cafaro A, Tripodi G, Pellottieri L, Mavilio D, Insalaco A, Croci S, Salvarani C, Gattorno M, Schena F. NET Proteomic Profiling Reveals New Pathways Potentially Implicated in Dendritic Cell-Mediated Inflammation in DADA2 Patients. *Journal of clinical immunology* 2025;**45**:106. <https://dx.doi.org/10.1007/s10875-025-01888-w>

7. Mou Z, Chen Y, Hu J, Hu Y, Zou L, Chen X, Liu S, Yin Q, Gong J, Li S, Mao S, Xu C, Jiang H. Icaritin inhibits the progression of urothelial cancer by suppressing PADI2-mediated neutrophil infiltration and neutrophil extracellular trap formation. *Acta pharmaceutica Sinica B* 2024;**14**:3916-30. <https://dx.doi.org/10.1016/j.apsb.2024.06.029>

8. Wang Y, Du C, Zhang Y, Zhu L. Composition and Function of Neutrophil Extracellular Traps. *Biomolecules* 2024;**14**<https://dx.doi.org/10.3390/biom14040416>

9. Divolis G, Synolaki E, Tringidou R, Tzouvelekis A, Boumpas DT, Skendros P, Galani IE. Transcriptomic analysis reveals shared deregulated neutrophil responses in COVID-19 and idiopathic pulmonary fibrosis. *Respiratory research* 2025;**26**:213. <https://dx.doi.org/10.1186/s12931-025-03180-2>

10. Huang J, Hong W, Wan M, Zheng L. Molecular mechanisms and therapeutic target of NETosis in diseases. *MedComm* 2022;**3**:e162. <https://dx.doi.org/10.1002/mco2.162>

11. Poli V, Pui-Yan Ma V, Di Gioia M, Broggi A, Benamar M, Chen Q, Mazitschek R, Haggarty SJ, Chatila TA, Karp JM, Zanoni I. Zinc-dependent histone deacetylases drive neutrophil extracellular trap formation and potentiate local and systemic inflammation. *iScience* 2021;**24**:103256. <https://dx.doi.org/10.1016/j.isci.2021.103256>

12. Zukas K, Cayford J, Serneo F, Atteberry B, Retter A, Eccleston M, Kelly TK. Rapid high-throughput method for investigating physiological regulation of neutrophil extracellular trap formation. *Journal of thrombosis and haemostasis : JTH* 2024;**22**:2543-54. <https://dx.doi.org/10.1016/j.jtha.2024.05.028>

13. Stewart AP, Loudon KW, Routledge M, Lee CYC, Trotter P, Richoz N, Gillman E, Antrobus R, McCaffrey J, Posner D, Conway Morris A, Karet Frankl FE, Clatworthy MR. Neutrophil extracellular traps protect the kidney from ascending infection and are required for a positive leukocyte dipstick test. *Science translational medicine* 2024;**16**:eadh5090. <https://dx.doi.org/10.1126/scitranslmed.adh5090>

14. Tseng KY, Huang YT, Huang YT, Su YT, Wang AN, Weng WY, Ke CL, Yeh YC, Wang JJ, Du SH, Gu ZQ, Chen WL, Lin CH, Tsai YH. Regulation of candidalysin underlies Candida albicans persistence in intravascular catheters by modulating NETosis. *PLoS pathogens* 2024;**20**:e1012319. <https://dx.doi.org/10.1371/journal.ppat.1012319>

15. Konwar S, Schroda S, Rogg M, Kleindienst J, Decker EL, Pohl M, Zieger B, Panse JP, Wang H, Grosse R, Schell C, Vidal S, Liu X, Gorzelanny C, Tschongov T, Haffner K. Thrombospondin-1 inhibits alternative complement pathway activation in antineutrophil cytoplasmic antibody-associated vasculitis. *The Journal of clinical investigation* 2025;<https://dx.doi.org/10.1172/JCI180062>

16. Remez-Gabay L, Vdovich O, Akria L, Kruzel-Davila E. Case Report: Anti-platelet factor 4 -mediated immunothrombosis in a patient with ANCA vasculitis - a shared mechanism of NETosis. *Frontiers in immunology* 2025;**16**:1567999. <https://dx.doi.org/10.3389/fimmu.2025.1567999>

17. Oh SA, Seol SI, Davaanyam D, Kim SW, Lee JK. Platelet-derived HMGB1 induces NETosis, exacerbating brain damage in the photothrombotic stroke model. *Molecular medicine* 2025;**31**:46. <https://dx.doi.org/10.1186/s10020-025-01107-7>

18. Huang H, Deng X, Wang Y, Shen S, Wang S, Hu M, Liu S, Su X, Li C, Li T, Lu Z, Cai W. Chronic Stress Exacerbates Cerebral Amyloid Angiopathy Through Promoting Neutrophil Extracellular Traps Formation. *Advanced science* 2024;**11**:e2404096. <https://dx.doi.org/10.1002/advs.202404096>

19. Azzouz D, Palaniyar N. How Do ROS Induce NETosis? Oxidative DNA Damage, DNA Repair, and Chromatin Decondensation. *Biomolecules* 2024;**14**<https://dx.doi.org/10.3390/biom14101307>

20. Inozemtsev V, Sergunova V, Vorobjeva N, Kozlova E, Sherstyukova E, Lyapunova S, Chernysh A. Stages of NETosis Development upon Stimulation of Neutrophils with Activators of Different Types. *International journal of molecular sciences* 2023;**24**<https://dx.doi.org/10.3390/ijms241512355>

21. Hayden H, Klopf J, Ibrahim N, Knobl V, Sotir A, Mekis R, Nowikovsky K, Eilenberg W, Neumayer C, Brostjan C. Quantitation of oxidized nuclear and mitochondrial DNA in plasma samples of patients with abdominal aortic aneurysm. *Free radical biology & medicine* 2023;**206**:94-105. <https://dx.doi.org/10.1016/j.freeradbiomed.2023.06.014>

22. Katayama H. Neutrophil Extracellular Traps Capturing SARS-CoV-2 in the Lung Tissue (Alveoli and Parenchyma) Cause Microthrombi　- A Strategy to Eliminate SARS-CoV-2 From the Circulation as Degraded Fibrin Clots. *Circulation reports* 2025;**7**:379-82. <https://dx.doi.org/10.1253/circrep.CR-24-0157>

23. Oliveira JD, Vieira-Damiani G, da Silva LQ, Leonardi GR, Vaz CO, Jacintho-Robison BC, Mazetto BM, de Paula EV, Monica FZ, Orsi FA. Impact of antiplatelets, anticoagulants and cyclic nucleotide stimulators on neutrophil extracellular traps (NETs) and inflammatory markers during COVID-19. *Journal of thrombosis and thrombolysis* 2025;**58**:199-209. <https://dx.doi.org/10.1007/s11239-024-03057-z>

24. Krinsky N, Sizikov S, Nissim S, Dror A, Sas A, Prinz H, Pri-Or E, Perek S, Raz-Pasteur A, Lejbkowicz I, Cohen-Matsliah SI, Almog R, Chen N, Kurd R, Jarjou'i A, Rokach A, Ben-Chetrit E, Schroeder A, Caulin AF, Yost CC, Schiffman JD, Goldfeder M, Martinod K. NETosis induction reflects COVID-19 severity and long COVID: insights from a 2-center patient cohort study in Israel. *Journal of thrombosis and haemostasis : JTH* 2023;**21**:2569-84. <https://dx.doi.org/10.1016/j.jtha.2023.02.033>

25. Sloan EE, Kmetova K, NaveenKumar SK, Kluge L, Chong E, Hoy CK, Yalavarthi S, Sarosh C, Baisch J, Walters L, Nassi L, Fuller J, Turnier JL, Pascual V, Wright TB, Madison JA, Knight JS, Zia A, Zuo Y. Non-criteria antiphospholipid antibodies and calprotectin as potential biomarkers in pediatric antiphospholipid syndrome. *Clinical immunology* 2024;**261**:109926. <https://dx.doi.org/10.1016/j.clim.2024.109926>

26. Zaiema S, Elwafa M, Hassan SGA, El Adwey R, Ghorab RMM, Galal R. Insight into antiphospholipid syndrome: the role and clinical utility of neutrophils extracellular traps formation. *Thrombosis journal* 2024;**22**:32. <https://dx.doi.org/10.1186/s12959-024-00598-4>

27. Zhu Y, Xia X, He Q, Xiao QA, Wang D, Huang M, Zhang X. Diabetes-associated neutrophil NETosis: pathogenesis and interventional target of diabetic complications. *Frontiers in endocrinology* 2023;**14**:1202463. <https://dx.doi.org/10.3389/fendo.2023.1202463>

28. Tao M, He Y, Li L, Li Y, Liao W, Nie H, Gao P. Identification and validation of immune-associated NETosis subtypes and biomarkers in anti-neutrophil cytoplasmic antibody associated glomerulonephritis. *Frontiers in immunology* 2023;**14**:1177968. <https://dx.doi.org/10.3389/fimmu.2023.1177968>

29. Zhao G, Cho CS, Liu H, Hwang Y, Si Y, Kim M, Deng Y, Zhao Y, Xue C, Guo Y, Chang L, Mizrak D, Yang B, Kang HM, Zhang J, Lee JH, Chen YE. Single-cell spatial transcriptomics unravels the cellular landscape of abdominal aortic aneurysm. *JCI insight* 2025;**10**<https://dx.doi.org/10.1172/jci.insight.190534>

30. Tsukui D, Kimura Y, Kono H. Pathogenesis and pathology of anti-neutrophil cytoplasmic antibody（ANCA）-associated vasculitis. *Journal of translational autoimmunity* 2021;**4**:100094. <https://dx.doi.org/10.1016/j.jtauto.2021.100094>

31. Xing L, Wu S, Xue S, Li X. A Novel Neutrophil Extracellular Trap Signature Predicts Patient Chemotherapy Resistance and Prognosis in Lung Adenocarcinoma. *Molecular biotechnology* 2025;**67**:1939-57. <https://dx.doi.org/10.1007/s12033-024-01170-1>

32. Xu X, Wang X, Zheng Z, Guo Y, He G, Wang Y, Fu S, Zheng C, Deng X. Neutrophil Extracellular Traps in Breast Cancer: Roles in Metastasis and Beyond. *Journal of Cancer* 2024;**15**:3272-83. <https://dx.doi.org/10.7150/jca.94669>

33. Snoderly HT, Boone BA, Bennewitz MF. Neutrophil extracellular traps in breast cancer and beyond: current perspectives on NET stimuli, thrombosis and metastasis, and clinical utility for diagnosis and treatment. *Breast cancer research : BCR* 2019;**21**:145. <https://dx.doi.org/10.1186/s13058-019-1237-6>
